# Plastome phylogenomics and biogeography of the subfam. Polygonoideae (Polygonaceae)

**DOI:** 10.3389/fpls.2022.893201

**Published:** 2022-10-05

**Authors:** Huajie Zhang, Xu Zhang, Yanxia Sun, Jacob B. Landis, Lijuan Li, Guangwan Hu, Jiao Sun, Bashir B. Tiamiyu, Tianhui Kuang, Tao Deng, Hang Sun, Hengchang Wang

**Affiliations:** ^1^ CAS Key Laboratory of Plant Germplasm Enhancement and Specialty Agriculture, Wuhan Botanical Garden, Chinese Academy of Sciences, Wuhan, China; ^2^ Center of Conservation Biology, Core Botanical Gardens, Chinese Academy of Sciences, Wuhan, China; ^3^ University of Chinese Academy of Sciences, Beijing, China; ^4^ Section of Plant Biology and the L.H. Bailey Hortorium, School of Integrative Plant Science, Cornell University, Ithaca, NY, United States; ^5^ BTI Computational Biology Center, Boyce Thompson Institute, Ithaca, NY, United States; ^6^ CAS Key Laboratory for Plant Diversity and Biogeography of East Asia, Kunming Institute of Botany, Chinese Academy of Sciences, Kunming, China; ^7^ Yunnan International Joint Laboratory for Biodiversity of Central Asia, Kunming Institute of Botany, Chinese Academy of Sciences, Kunming, China

**Keywords:** dispersal routes, phylogenomic, plastomes, Polygonoideae, biogeography

## Abstract

Polygonaceae has a complex taxonomic history, although a few studies using plastid or nuclear DNA fragments have explored relationships within this family, intrafamilial relationships remain controversial. Here, we newly sequenced and annotated 17 plastomes representing 12 genera within Polygonaceae. Combined with previously published data, a total of 49 plastomes representing 22/46 Polygonaceae genera and 16/20 Polygonoideae genera were collected to infer the phylogeny of Polygonaceae, with an emphasis on Polygonoideae. Plastome comparisons revealed high conservation within Polygonoideae in structure and gene order. Phylogenetic analyses using both Maximum Likelihood and Bayesian methods revealed two major clades and seven tribes within Polygonoideae. BEAST and S-DIVA analyses suggested a Paleocene origin of Polygonoideae in Asia. While most genera of Polygonoideae originated and further diversified in Asia, a few genera experienced multiple long-distance dispersal events from Eurasia to North America after the Miocene, with a few dispersal events to the Southern Hemisphere also being detected. Both ancient vicariance and long-distance events have played important roles in shaping the current distribution pattern of Polygonoideae.

## Introduction

Polygonaceae Juss. (Caryophyllales) contains approximately 46 genera and 1200 species with morphological disparity, with life forms varying from herbs, lianas, woody vines, shrubs to trees (Kubitzki et al., 1990; Sanchez et al., 2009; Burke and Sanchez, 2011; Schuster et al., 2015). Polygonaceae is widely distributed around the world, mainly in temperate North America, Europe and Southeast Asia, but species can also be found in South America, the Caribbean, Africa and Australasia (Frye and Kron, 2003; Burke and Sanchez, 2011; Sanchez et al., 2011). Classification within Polygonaceae, specifically the definitions of different subfamilies or genera, has long been controversial (Burke and Sanchez, 2011; Sanchez et al., 2011; Schuster et al., 2013). In previous studies, subfamilies were primarily differentiated based on morphological characters, e.g., the presence or absence of ocreas, tepal arrangement and habitat, with two to four subfamilies having been proposed (Meisner, 1856; Jaretzky, 1925). Meisner (1856) suggested four subfamilies including Polygonoideae Eaton, Eriogonoideae Arn, Brunnichioideae Meisn. and Symmerioideae Meisn. Later, a proposal of three subfamilies including Polygonoideae, Eriogonoideae and Coccoloboideae Luerss. was suggested (Perdrigeat, 1900). Afterwards Coccoloboideae was merged with Polygonoideae by Jaretzky (1925). Generally, the recognition of Polygonoideae and Eriogonoideae as subfamilies of Polygonaceae has been widely accepted. Typically, Polygonoideae is characterized by possessing ocreae, while Eriogonoideae is characterized by lacking ocreae (Jaretzky, 1925).

In general, Polygonoideae is recognized to comprise 20 genera (Schuster et al., 2015). The subfamily is further divided into different tribes step by step (Sanchez and Kron, 2008; Sanchez et al., 2011). Sanchez and Kron (2008), using sequences of three chloroplast DNA segments and *LEAFY*, revealed three tribes: Persicarieae Dumort., Rumiceae Dumort., and Polygoneae Rchb. Galasso et al. (2009) divided the subfamily into four tribes including Persicarieae, Rumiceae, Polygoneae and Fagopyreae Yonke. based only on *rbcL* sequences. Afterwards, Sanchez et al. (2011) proposed five tribes Persicarieae, Rumiceae, Polygoneae, Fagopyreae and Calligoneae based on *matK*, *ndhF* and *rbcL* sequences with a more comprehensive sampling. More recently, an updated phylogenetic analysis suggested seven tribes with two tribes, Oxygoneae T.M.Schust. & Reveal and Pteroxygoneae T.M.Schust. & Reveal, added using *matK*, *trnL-F* and ITS sequences (Schuster et al., 2015). Polygonoideae has a worldwide distribution, covering North America, South America, Europe, Asia, Africa and Oceania. Previous biogeographical studies revealed that taxa displaying intercontinental disjunction usually have an Asian origin, followed by long-distance dispersal and vicariance (e.g., Givnish and Renner, 2004; Yoder and Nowak, 2006; Nie et al., 2012; Fritsch et al., 2015; Yang et al., 2018). Notably, 17 Polygonoideae genera can be found in Asia; however, up till now, studies investigating the area of origin and dispersal of the subfamily are absent and thus limiting a comprehensive understanding of the evolutionary history of Polygonoideae.

Whole plastome sequencing is a cost-effective method that has been widely applied to resolve phylogenetic relationships at different taxonomic levels (Dong et al., 2020; Jung et al., 2021; Wen et al., 2021). Recently, such approaches have been applied to investigate the phylogenetic relationships of genera within Polygonaceae (Song et al., 2020; Zhou et al., 2020; Yang et al., 2021; Zhang et al., 2021). However, a comprehensive analysis, including plastome structure, phylogenomics and biogeography of Polygonoideae is lacking. In this study, we newly assembled 17 Polygonaceae plastomes to explore phylogenetic relationships and trace the origins of Polygonoideae along with previously published plastome sequences. The main objectives of this study are to (1) analyze and compare plastomes to track plastome evolution within Polygonoideae, and (2) elucidate the phylogeny and the biogeographic history of Polygonoideae to gain insights into the origin and the drivers of diversification of the subfamily.

## Materials and methods

### Taxon sampling, DNA extraction and sequencing

A total of 49 plastomes representing 22 genera of Polygonaceae were sampled, covering most genera of Polygonoideae and six genera from other subfamilies of Polygonaceae as of the most recent treatment of Schuster et al. (2015). Five individuals from three genera in Plumbaginaceae were selected as outgroups. Seventeen samples of Polygonaceae were newly sequenced and collected from around the world (**Table S1**). Fresh leaves were collected in silica-gel for DNA extraction. Voucher specimens were deposited at the Wuhan Botanical Garden (**Table S1**). All plastome sequences used in this study (including downloaded and newly sequenced) are listed in **Table 1**. Genomic DNA was extracted with a modified CTAB method (Li et al., 2013). DNA concentration was measured using the Qubit^®^ DNA Assay Kit with a Qubit^®^ 2.0 Fluorometer (Life Technologies, CA, USA). For library preparation, 1.5 µg of DNA per sample was used and fragmented by sonication to a size of 350 bp. Generated DNA fragments were end polished, A-tailed, and ligated with full-length adapters for Illumina sequencing with further PCR amplification. Finally, purified PCR products were analyzed for size distribution with an Agilent2100 Bioanalyzer and quantified using real-time PCR. Constructed libraries were sequenced on an Illumina HiSeq using 150 bp paired-end reads with an insert size around 350 bp. A minimum of 2 GB of raw sequencing data was generated for each accession.

**Table 1:** Summary of major characteristics of all 54 chloroplast genome sequences, including sequence length (bp), numbers of genes, GC content (%), and GenBank accession number.

Family	genus	species	whole genome						LSC length	SSC length	IRs length	GenBank accessions No.
			Length	GC (%)	No. of Genes	No. of PCGs	No. of rRNA	No. of tRNA				
Polygonaceae	*Rheum*	*Rheum palmatum*	161541	37.3	131	79	8	37	86518	13111	30956	NC027728
		*Rheum franzenbachii*	161688	37.4	131	79	8	37	86946	12784	30979	MN564923
		*Rheum racemiferum*	161682	37.4	131	79	8	37	87152	12822	30854	MN564928
		*Rheum pumilum*	161749	37.3	131	79	8	37	86997	12806	30973	MN564927
		*Rheum acuminatum*	161306	37.4	131	79	8	37	86145	13169	30996	MN564922
	*Oxyria*	*Oxyria digyna*	160698	37.5	131	79	8	37	85749	13171	30889	MN564931
		*Oxyria sinensis*	160404	37.5	131	79	8	37	85501	13133	30885	NC032031
	*Rumex*	*Rumex crispus*	161292	37.4	131	79	8	37	87213	13011	30534	MN564930
		*Rumex nepalensis*	159110	37.5	129	78	8	37	84810	13044	30628	MT457825
		*Rumex japonicus*	159292	37.5	130	78	8	37	85028	13006	30629	MN720269
		*Rumex acetosa*	160269	37.2	130	79	8	36	86135	13128	30503	NC042390
		*Rumex hypogaeus*	159413	37.5	128	79	8	36	85610	13109	30347	NC050054
	*Muehlenbeckia*	Muehlenbeckia complexa	163362	37.4	130	79	8	36	88223	13463	30838	MZ997424
		Muehlenbeckia australis	163484	37.4	131	79	8	37	88166	13486	30916	MG604297
	***Homalocladium***	***Homalocladium platycladum***	163202	37.3	130	78	8	37	87820	13538	30922	**OK661159**
	***Fallopia***	***Fallopia aubertii***	162393	37.6	131	78	8	37	87279	13394	30860	**OK661149**
	***Reynoutria***	*Reynoutria japonica*	163183	37.5	130	79	8	36	87905	13560	30859	MW411186
		***Reynoutria japonica***	163371	37.5	132	78	8	37	87571	13558	31121	OK661148
		*Reynoutria sachalinensis*	163485	37.5	130	79	8	37	87703	13566	31108	NC047446
	***Pleuropterus***	***Pleuropterus multiflorus***	163496	37.5	131	78	8	37	88112	13572	30906	**OK661155**
	*Atraphaxis*	*Atraphaxis bracteata*	164264	37.4	129	77	7	37	88854	13520	30945	MW363800
		*Atraphaxis irtyschensis*	164192	37.5	148	79	10	54	88877	13485	30915	MG878984
	***Polygonum***	***Polygonum aviculare***	163461	37.5	131	79	10	37	88021	13306	31067	**OK661156**
	*Calligonum*	*Calligonum leucocladum*	161279	37.5	131	79	8	37	86836	13361	30541	NC053260
		*Calligonum gobicum*	161375	37.5	131	79	8	37	86915	13356	30552	NC049139
		*Calligonum aphyllum*	161251	37.5	131	79	8	37	86853	13346	30526	NC049137
		*Calligonum arborescens*	162004	37.5	131	79	8	37	87629	13323	30526	NC049140
		*Calligonum jeminaicum*	162525	37.5	131	79	8	37	88160	13319	30528	NC049146
	***Pteroxygonum***	***Pteroxygonum denticulatum***	162897	37.4	131	78	8	37	88024	13167	30853	**OK661160**
	*Fagopyrum*	*Fagopyrum dibotrys*	159320	37.9	122	79	8	37	84422	13264	30817	NC037705
		*Fagopyrum tataricum*	159272	37.9	121	78	8	38	84397	13241	30817	NC027161
		*Fagopyrum esculentum subsp ancestrale*	159599	38	131	79	8	37	84885	13344	30685	NC010776
		*Fagopyrum leptopodum*	159337	37.8	130	79	8	37	84429	13226	30841	MW017634
		*Fagopyrum luojishanense*	159265	37.8	131	79	8	37	84431	13094	30870	NC037706
	***Persicaria***	***Persicaria orientalis***	159016	38.2	132	78	8	37	83585	13153	31139	OK661150
		***Persicaria filiforme***	159740	37.8	132	78	8	37	84444	13050	31123	OK661145
		***Persicaria perfoliata***	160735	38	132	78	8	37	85438	12927	31185	OK661161
		***Persicaria chinense var procumbens***	159074	38	131	78	8	38	84378	12894	30901	OK661147
		***Persicaria chinense***	159073	38	131	78	8	37	84307	12901	30901	OK661146
	***Bistorta***	*Bistorta vivipara*	158852	38	129	78	8	37	83797	13161	30947	MT066039
		***Bistorta macrophylla***	158885	38	131	78	8	37	83818	13161	30953	OK661158
	***Koenigia***	***Koenigia islandica***	155739	37.2	130	77	8	36	81503	13078	30579	**OK661154**
		***Koenigia forrestii***	156844	37.3	131	78	8	37	82565	13089	30595	**OK661157**
	***Coccoloba***	***Coccoloba unifera***	169360	36.6	131	78	8	37	92653	14067	31320	**OK661151**
	***Triplaris***	***Triplaris americana***	171340		132	78	8	37	95500	13678	31081	**OK661152**
	*Antigonon*	Antigonon leptopus (partial genome)	132199	37.2	/	/	/	/	/	/	/	MH286313
	*Afrobrunnichia*	*Afrobrunnichia erecta* (partial genome)	170974	37.1	/	/	/	/	88058	13654	34631	MH286316
	*Symmeria*	Symmeria paniculata (partial genome)	162501	38.3	/	/	/	/	86990	13437	31073	MH286353
	***Ruprechtia***	***Ruprechtia albida***	157255	37.8	129	78	8	36	86439	18622	26097	**OK661153**
Plumbaginaceae (outgroups)	*Limonium*	*Limonium aureum*	154661	37.1	130	78	8	37	84545	12980	28568	NC045399
		*Limonium sinense*	174033	36.7	132	79	8	37	96128	13517	32194	MN599096
		*Limonium tenellum*	150515	36.7	124	78	6	36	84634	23753	21064	NC041279
	*Ceratostigma*	*Ceratostigma willmottianum*	164999	37.5	127	77	8	37	89454	13491	31027	NC041261
	*Plumbago*	*Plumbago auriculata*	168765S	37.2	132	79	8	37	91912	13331	31761	NC041245

Sequence newly obtained are indicated by bold font words in “genus” and “species” line, genera were newly sequenced in this study are indicated by bold font words in “GenBank accessions No.” line.

### Plastome assembly, annotation and comparison

Raw sequencing reads were filtered using Trimmomatic v0.39 (Bolger et al., 2014) to remove adapters, low quality and unidentified nucleotides reads with the following parameters: LEADING=5, TRAILING=5, SLIDINGWINDOW=4:5, MINLEN=25. Clean reads were then *de novo* assembled using NOVOPlasty v4.3.1 (Dierckxsens et al., 2017) with the RUBP sequences as the seed for chloroplast assembly. The plastome sequence of *Rheum palmatum* (NCBI accession NC027728) was set as the reference. Assembled plastomes were annotated with PGA (Qu et al., 2019), again using *R. palmatum* (NCBI accession NC027728) as the reference. The preliminary annotated sequences were imported into Geneious v.9.0.2 to check start/stop codons and intron/exon boundaries (Kearse et al., 2012). Newly determined plastome sequences were submitted to NCBI (National Center for Biotechnology information) GeneBank (**Table 1**).

To detect inverted repeat (IR) expansion or contraction of plastomes and compare the boundary genes of the SC and IR among Polygonoideae, we chose *R. palmatum* (NCBI accession NC027728) as the reference and visualized the borders of the LSC, SSC, and IR in 17 genera in Polygonoideae using IRscope (Amiryousefi et al., 2018). Among the 17 plastomes, eight were downloaded from NCBI and nine accessions were newly sequenced in this study.

### Phylogenetic analyses

To explore the phylogenetic relationships of Polygonoideae, a total of 54 plastomes representing 23 Polygonaceae genera were included in the phylogenomic analysis. Among these, 37 plastomes were download from Genebank and 17 plastomes were newly sequenced. Five species of Plumbaginaceae were selected as outgroups, including three species of *Limonium*, one species of *Plumbago* and one species of *Ceratostigma* (**Table 1**). For each plastome, we extracted 74 shared PCGs and aligned them with the codon-aware program MACSE v2.03 (Ranwez et al., 2018) followed by manual examination and adjustment in Mega X (Kumar et al., 2018). Alignments of PCGs were concatenated into a super-matrix with PhyloSuite v1.2.2 (Zhang et al., 2019a). The complete plastome nucleotide sequences, including only one copy of the IR regions were also used for the phylogenetic analyses. Both maximum-likelihood (ML) and Bayesian inference (BI) analyses were conducted for phylogenetic inference. For the ML analysis, RAxML v8.2.12 (Stamatakis, 2014) was used with the general time reversible model for nucleotide substitution, the gamma model of rate heterogeneity (GTR+G), and 500 rapid bootstrap replicates. Bayesian analyses were conducted with MrBayes v3.2.7 (Huelsenbeck and Ronquist, 2001). The best-fit model was calculated with ModelTest-NG (Darriba et al., 2020) under the Bayesian information criterion (BIC). Two runs with four Markov chains were applied with 2,000,000 generations from a random starting tree with sampling every 500 generations. The initial 25% of sampled trees were discarded as burn-in and the remaining trees were used to construct a majority-rule consensus tree and calculate the posterior probability. Bootstrap support (BS) and posterior probability (PP) were used to measure the support of the generated phylogenetic trees. Additionally, since we were not able to generate plastomes of *Oxygonum* and *Knorringia* in Polygonoideae, three chloroplast fragments (*matK*, *trnL* and *rbcL)* were extracted from the plastomes used here and combined with available data online to explore their phylogenetic positions (**Table S3**). A tree based on our results combined with previous studies were generated to exhibit a comprehensive phylogeny of Polygonoideae. The final phylogenetic topologies were viewed in FigTree v1.3.1 (Rambaut, 2009).

### Estimation of divergence time

BEAST v1.10.4 (Drummond and Rambaut, 2007) was used to estimate the divergence time between lineages of Polygonaceae using the concatenated three chloroplast loci data matrix for inclusion of the largest number of genera. We chose the GTR+G substitution model, with a relaxed molecular clock model and Yule process as the tree prior. According to previous studies (Manchester and O’leary, 2010; Schuster et al., 2013; Yao et al., 2019), two fossil calibrations and two secondary calibrations were used. A detailed discussion of the fossil calibrations is provided in **Supplementary Methods S1**. The crown age of Polygonaceae was set to an age range of 72.1-66.0 Ma with a lognormal calibration prior (Manchester and O’leary, 2010). The crown age of *Muehlenbeckia* was set to an age range of 22.0–19.0 Ma with a lognormal calibration prior (Pole, 1992; Schuster et al., 2013). The crown age of Plumbaginaceae was set to a mean age of 60.0 Ma with a SD of 3.0 Ma and the crown age of all included species was set to a mean age of 91.8 Ma with a SD of 0.5 Ma (Yao et al., 2019). Both secondary calibration priors were set as a normal distribution. The MCMC was run for 1 x 10^9^ generations, sampling every 10,000 generations. The convergence of the two runs and stationarity of the chains were checked in Tracer v1.7 (Rambaut et al., 2018), with a sufficient effective sample size (ESS) > 200 for all relevant parameters. The first 25% trees were discarded as burn-in, and a maximum clade credibility tree with mean heights reported for node heights and 95% highest posterior density intervals (95% HPDs) was generated with TreeAnnotator v1.10.4 (Suchard et al., 2018). FigTree v1.3.1 was used for visualizing the resulting phylogenetic tree (Rambaut, 2009).

### Biogeographic analysis

We collected species distribution data of Polygonoideae from monographs, regional floras and online databases such as GBIF (https://www.gbif.org) and JSTOR Global Plants (https://plants.jstor.org/). Based on the distribution data and related geological history, we defined seven biogeographic areas: (A) North America, (B) South America, (C) Europe, (D) Asia, (E) Africa, and (F) Oceania. We used BioGeoBEARS as implemented in RASP 4.0 (Matzke, 2014; Yu et al., 2015) to explore the ancestral area of Polygonoideae with a Statistical Dispersal-Vicariance Analysis (S-DIVA) (Yu et al., 2010) and a condensed tree derived from the BEAST analysis (outgroups were excluded).

## Results

### Genome assembly and plastomes features

**Figure 1:**
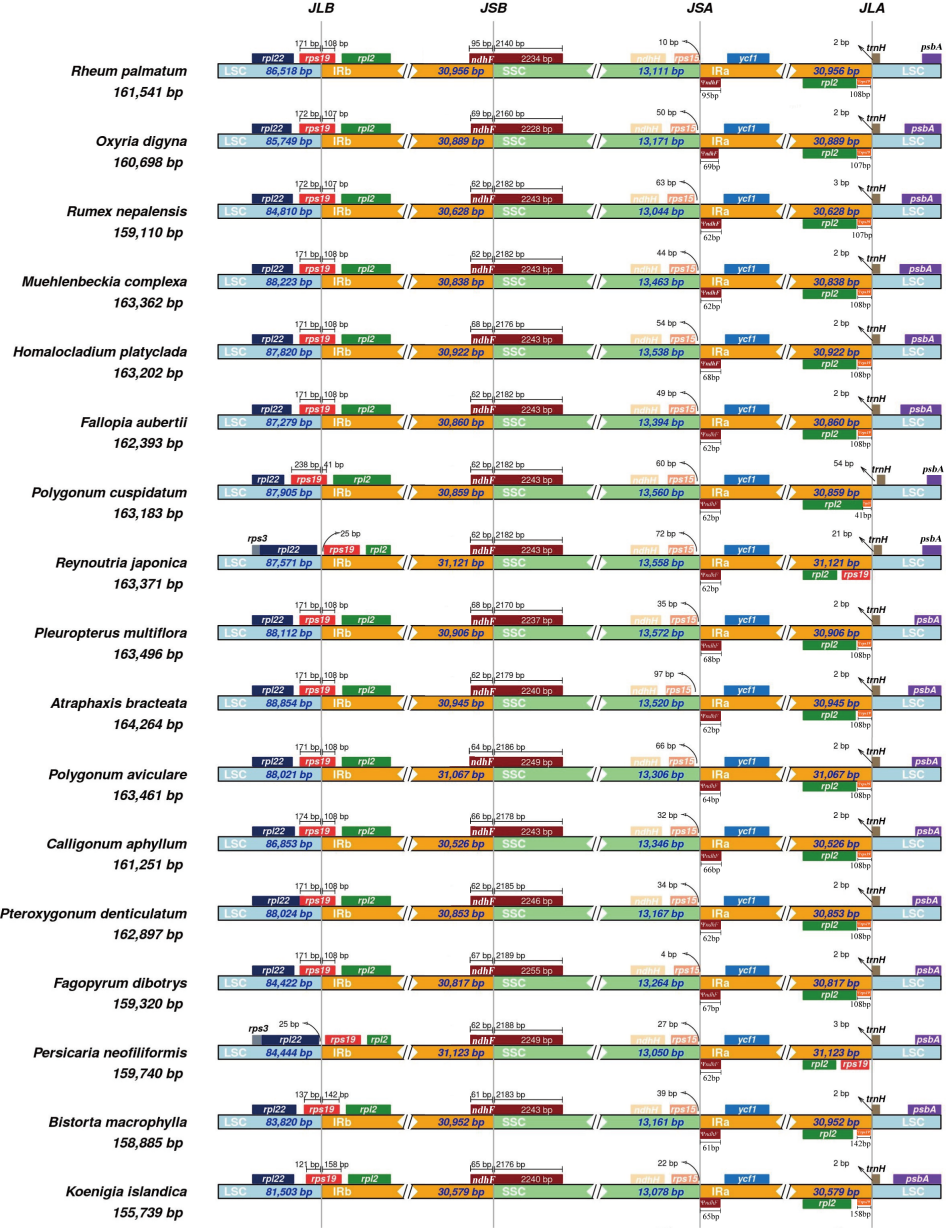
The IR/SSC borders in the plastomes of 17 genera of Polygonoideae. JLB, JSB, JSA and JLA denote the junction sites of LSC/IRb, SSC/IRb, SSC/IRa and LSC/IRa respectively.

Illumina sequencing generated a total of 12,997,916- 20,520,168 paired-end clean reads for each species (**Table S1**). The mean sequencing coverage of the observed plastomes ranged from 209× to 3,956× (**Table S1**). The 17 newly sequenced plastomes, ranging in size from 155,739 to 171,340 bp, displayed a typical quadripartite structure and similar gene order consisting of a LSC (81,503-95,500 bp), SSC (12,806- 18,622 bp), and two IRs (IRa and IRb; 26,097-31,320 bp) (**Table 1**). The plastomes contained 77-79 protein coding genes, seven to 10 rRNA genes and 36-38 tRNA genes arranged in the same order. Plastome comparisons revealed no clear expansions or contractions in the IR regions (**Figure 1**). The LSC/IR boundaries of 17 Polygonoideae plastomes were all located at the *rps19* and *trnH*-GUG, while the SSC/IR boundaries were all located at *ndhF* and *rps15* (**Figure 1**).

### Evolutionary rates of plastomes

Nucleotide diversity (pi) and Watterson’s theta estimator (θ) of the 74 shared PCGs were calculated in DNAsp v6 to assess the sequence divergence level of Polygonoideae (Rozas et al., 2017). Among the 74 PCGs, pi values ranged from 0.0066 (ndhB) to 0.0991 (ndh*F*) (**Figure S1A**, **Table S2**), with highly divergent genes identified as having pi > 0.08. Four genes were detected as highly divergent including *ccsA*, *matK*, *ndhF* and *rps15* (**Figure S1A**, **Table S2**), which can be potential molecular markers for phylogenetic analyses.

### Phylogenetic relationships

**Figure 2:**
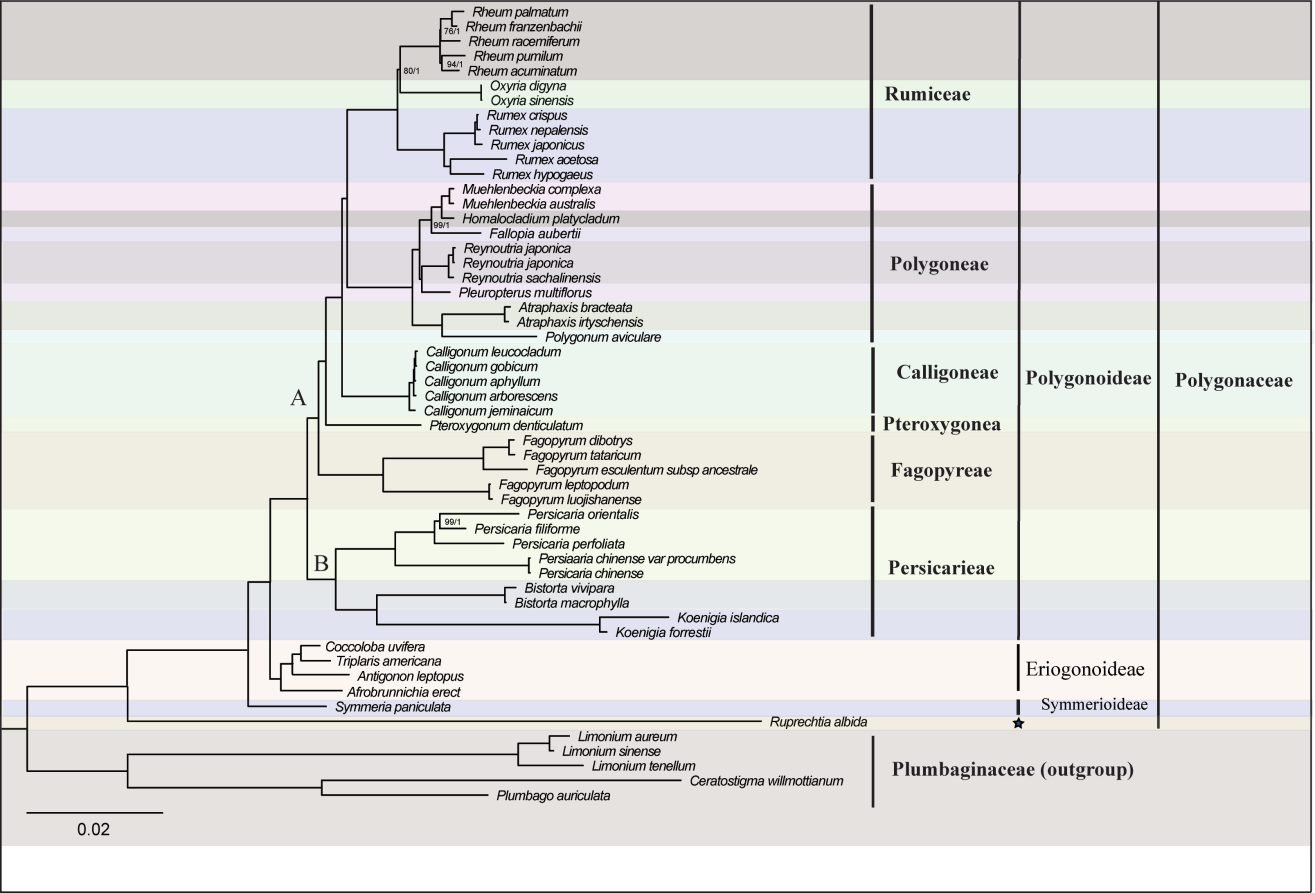
Phylogenetic tree of 54 taxa using maximum likelihood (ML) and Bayesian inference (BI) based on 74 shared genes. Maximum likelihood bootstrap values (BS) and posterior probabilities (PP) are shown at nodes. Branches with no values listed have 100% BS and PP of 1.0. Pentagram represents uncertain classification treatment.

**Figure 3:**
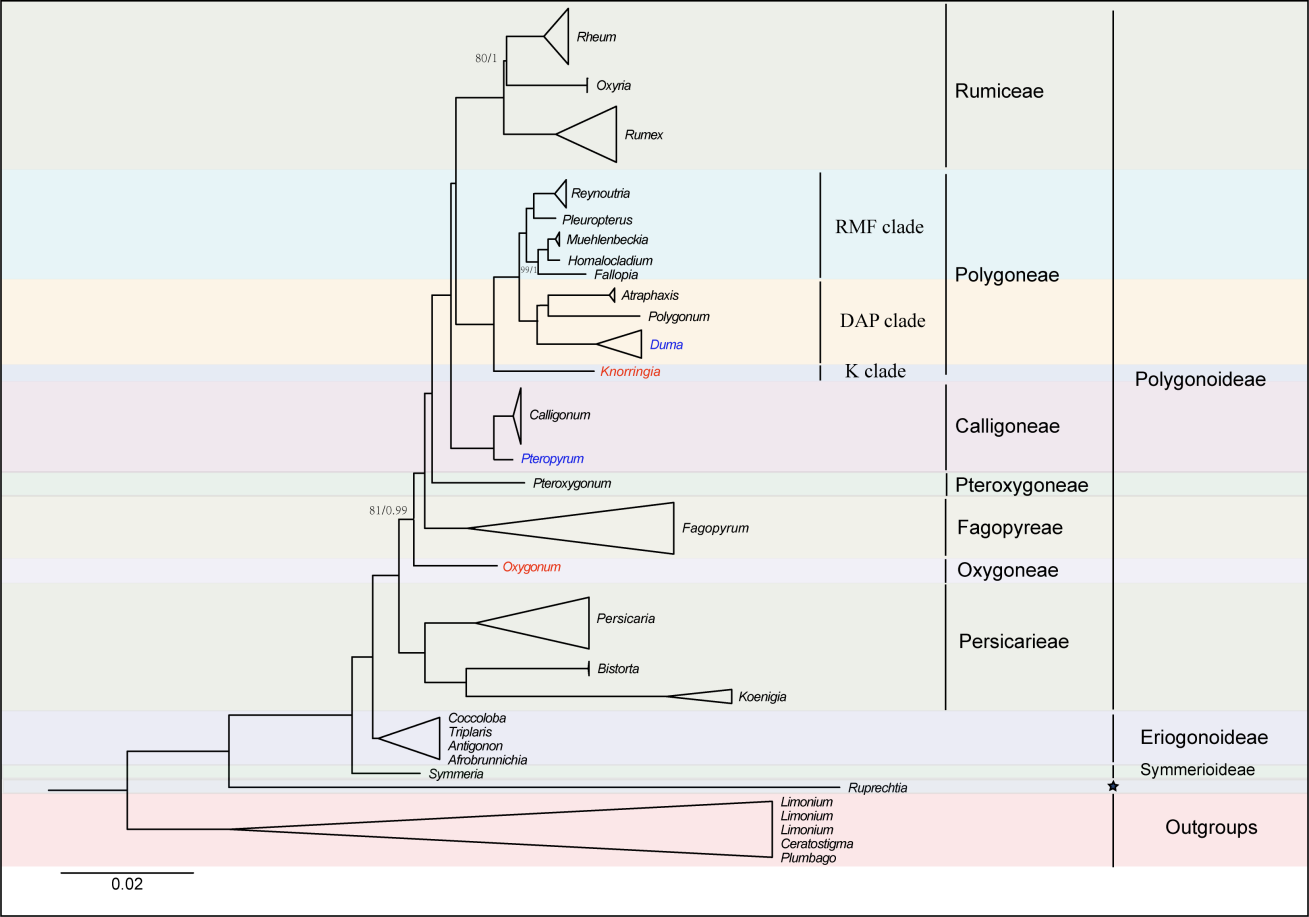
An update phylogenetic tree based on 74 shared PCGs and chloroplast fragments, with positions of species in red being generated from chloroplast fragments. Maximum likelihood bootstrap values (BS) and posterior probabilities (PP) are shown at nodes. Branches with no values listed have 100% BS and PP of 1.0 except for species with blue. Positions of species with blue were generated from previous studies (Schuster et al., 2011, 2015). Pentagram represents uncertain classification treatment.

The alignment matrix of the 74 PCGs was 73,025 bp in length, with 14,430 Parsimony-informative sites and 22,084 variable sites. The analyses of the concatenated matrix and the complete plastome nucleotide sequences matrix generated identical topologies at every node (**Figures 2**, **Figure S2**). The phylogenetic analysis with three chloroplast DNA fragments also generated identical relationships although some clades were weakly supported (**Figure S3**, **Table S3**). Hence an updated phylogeny was generated by combing all of the above phylogenetic trees (**Figure 3**). Generally, the phylogenetic analyses revealed Polygonoideae as monophyletic and composed of two subclades, clade A and clade B (**Figures 2**, **3**). Clade A consists of Rumiceae Dumort., Polygoneae Rchb., Calligoneae C. A. Mey., Pteroxygonea T.M.Schust. & Reveal and Fagopyreae Yonek. While Clade B contains only the tribe Persicarieae Dumort. Rumiceae consists of three genera: *Rheum*, *Oxyria* and *Rumex*. Polygoneae consists of seven genera. Calligoneae, Pteroxygonea and Fagopyreae each consist of one genus. Persicarieae consists of three genera. Both the 74-PCGs tree and the tree based on three chloroplast loci revealed that Eriogonoideae is not monophyletic (**Figures 2**, **S2**).

### Time estimation and biogeographic analysis

**Figure 4:**
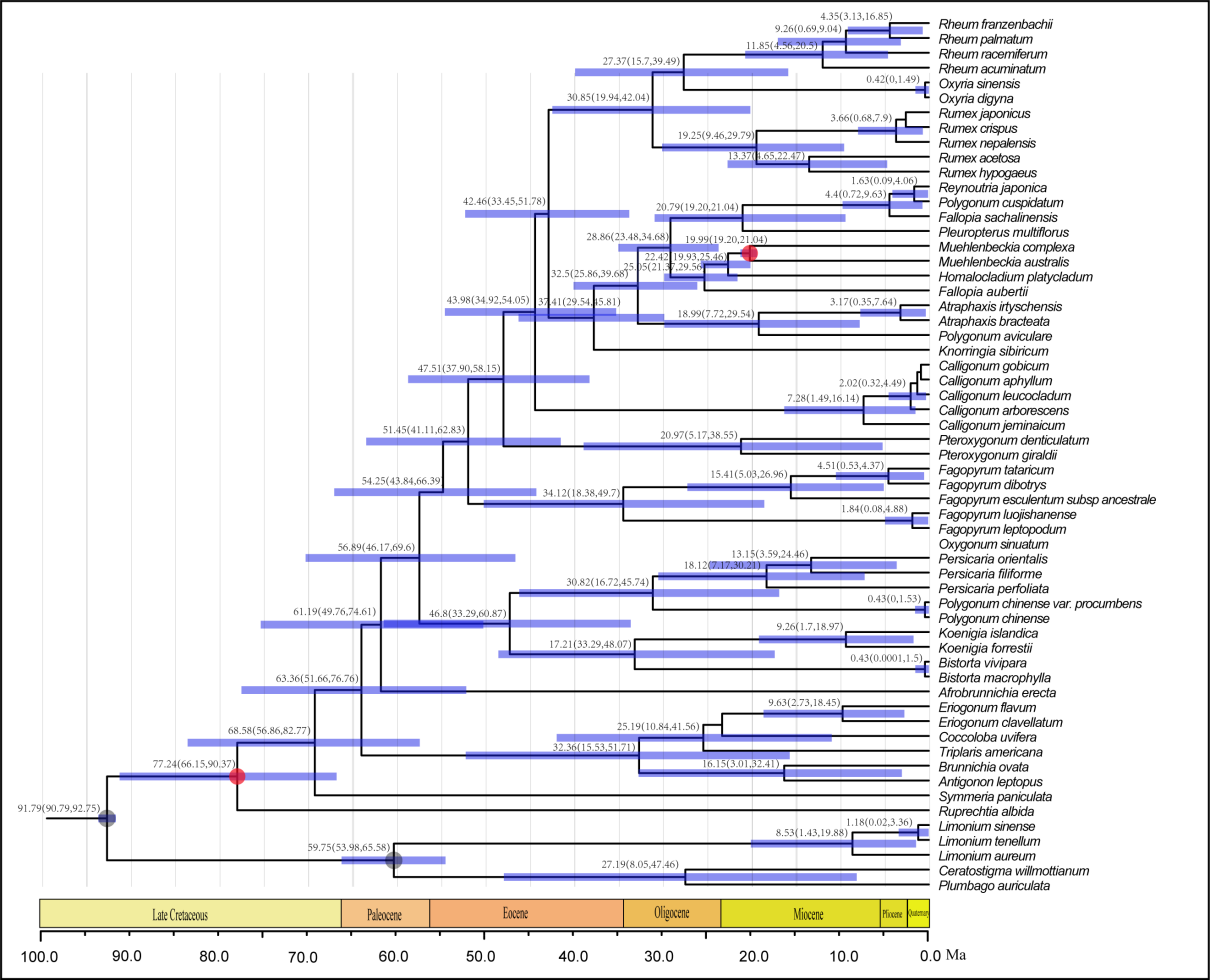
BEAST-derived chronogram of Polygonaceae based on three chloroplast fragments with two fossil calibration (red circles) and two second calibration point (gray circles), fossils’ information were provided in **Supplementary Methods S1**. Numbers above the tree branches represented mean divergent ages and 95% confidence interval of each node. Blue bars indicate the 95% highest posterior density (HPD) confidence intervals for node ages (Ma).

Results of the dating analyses suggested that Polygonoideae diverged from other members of Polygonaceae during the late Paleocene (54.9 Ma, 95% HPD: 45.2-65.6 Ma). The split of the two major clades in Polygonoideae occurred in the early Eocene (50.45 Ma, 95% HPD: 41.6-60.1 Ma). In Clade B (Persicarieae), *Persicaria* and the other two genera (*Bistora* and *Koenigia*) diverged during the Eocene (42.5 Ma, 95% HPD: 32.3-52.4 Ma) (**Figure 4**). In Clade A, *Fagopyreae* and the remaining tribes also diverged during the Eocene (46.8 Ma, 95% HPD: 37.6-55.6 Ma); *Pteroxygonea* diverged during the Eocene (43.5 Ma, 95% HPD: 34.9-51.6 Ma) and *Calligoneae* diverged during the late Eocene (39.5 Ma, 95% HPD: 32.1-47.2 Ma). The divergence of Rumiceae and Polygoneae was estimated to have occurred during the late Eocene (37.1 Ma, 95% HPD: 29.9-44.3 Ma). In Polygoneae, the included genera diverged during the Oligocene (30.7 Ma, 95% HPD: 25.2-36.9 Ma) (**Figure 4**). In Rumiceae, genera diverged during the late Oligocene (25.9 Ma, 95% HPD: 16.8-34.4 Ma); *Rheum* and *Oxyria* diverged during the early Miocene (22.4 Ma, 95% HPD: 13.3-31.7 Ma) (**Figure 4**).

**Figure 5:**
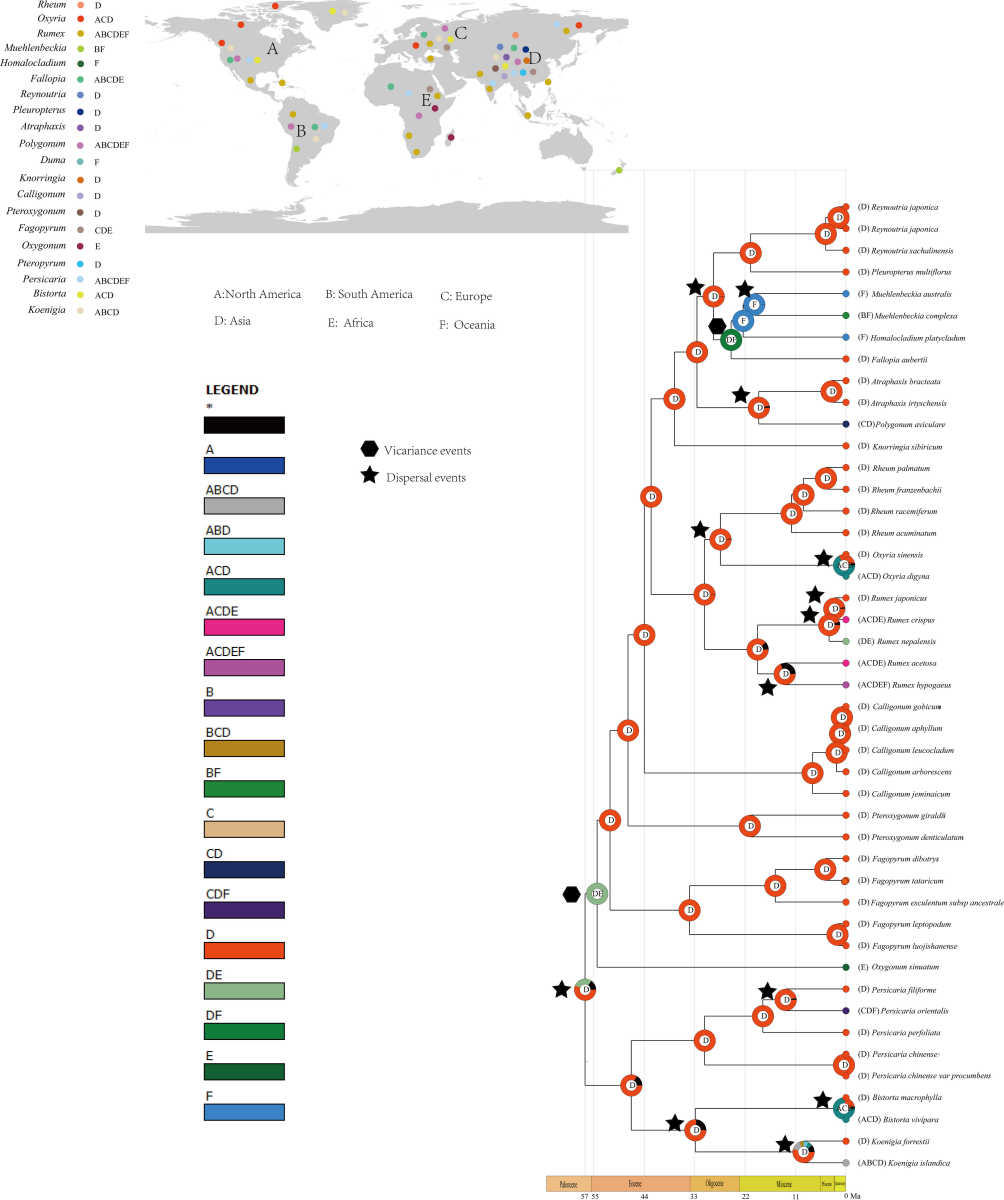
Ancestral area reconstruction of Polygonaceae. Figure of upper left shows the distribution of each genus.

Results from the biogeographic analysis suggest that the ancestor of Polygonoideae is likely from Asia. Much of the lineage of Polygonoideae was reconstructed with an Asian origin (**Figure 5**). A total of two vicariance and 26 dispersal events were detected (**Figure 5**). Migrations to Europe and North America were common and most occurred after the Miocene. Dispersals to South America and Oceania were also detected, with one vicariance event occurring in Asia and Africa during the Eocene, while another vicariance event in this area occurred during the Oligocene.

## Discussion

### Phylogenetic analyses

The monophyly of Polygonaceae is supported by the analyses in the current study and those in previous studies (Sanchez and Kron, 2008; Sanchez et al., 2009; Schuster et al., 2015). The definition of different subfamilies within Polygonaceae based on morphological characters has been controversial over the past 200 years (e.g., Meisner, 1856; Perdrigeat, 1900; Jaretzky, 1925). However, the subfamily Polygonoideae and Eriogonoideae have been widely supported by subsequent molecular analyses (Frye and Kron, 2003; Galasso et al., 2009; Schuster et al., 2015). The monophyly of Polygonoideae is confirmed in the current study (**Figures 2**, **S2**) and our results show Polygonoideae consists of seven clades, corresponding to the seven tribes defined by Schuster et al. (2015). Oxygoneae was previously shown as the earliest-diverging clade in Schuster et al. (2015), while our analyses show Persicarieae is the earliest-diverging clade of Polygonoideae, followed by Oxygoneae (**Figures 2**, **S2**, **S3**). The relationships among Oxygoneae, Fagopyreae, Pteroxygoneae, Calligoneae, Polygoneae and Rumiceae in the current analyses are identical with those revealed by Schuster et al. (2015). Our analyses also identified similar clades within Polygoneae as suggested previously (Schuster et al., 2015). Notably, *Pleuropterus* is included here for the first time in a phylogenetic analysis of Polygonoideae. Unlike previous studies (Sanchez et al., 2009; Sanchez et al., 2011; Schuster et al., 2013; Schuster et al., 2015), the current analyses resolved relationships within Rumiceae; *Rheum* and *Oxyria* form a sister relationship with 80/1 (bootstrap and posterior probability) support value. Although several genera including *Pteropyrum* and *Parogonum* are absent from the phylogenetic analyses of Polygonoideae here, the relationships among different Polygonoideae tribes are well resolved with high support. Despite our sampling of Eriogonoideae being inadequate for phylogenetic analyses of the subfamily, a nonmonophyletic signal for Eriogonoideae is revealed (**Figures 2**, **S2**). The nonmonophyly of Eriogonoideae has also been reported in Sanchez et al. (2009). Both *Symmeria* and *Ruprechtia* fall outside of the large clade comprised by Polygonoideae and some Eriogonoideae genera (**Figures 2**, **S2**), suggesting dividing Polygonaceae into three or more subfamilies is reasonable, which needs to be confirmed by more adequate sampling and sequencing in the future.

### Divergence times and biogeography

Divergence time estimation and S-DIVA analysis revealed an Asia origin of Polygonoideae during the Eocene, with subsequent migrations primarily to Europe and North America (**Figure 5**). Asia was inferred as the ancestral distribution area of all Polygonoideae tribes except for Oxygoneae (**Figure 5**). Previous studies of taxa displaying intercontinental disjunction also found these taxa more often originated in Asia, especially in the Qinghai-Tibet Plateau (QTP) region (Morley, 2003; Nie et al., 2013; Sun et al., 2017; Zhang et al., 2019b). Some taxa, e.g., *Carex* (Cyperaceae)*, Urtica* (Urticaceae) and Balsaminaceae, have similar distributions as Polygonoideae (**Figure 5**), following dispersal and vicariance events after origins in Asia, which have played key roles in shaping current distribution patterns (Yuan et al., 2004; Huang et al., 2019; Martín‐Bravo et al., 2019). Although the breakup of the Gondwanan supercontinent may have resulted in a disjunct distribution pattern in lineages (Raven and Axelrod, 1974; Conti et al., 2002), many lineages are found to originate more recently (**Figure 5**), indicating the possibility of long-distance dispersal occurring during their evolutionary histories. All dispersal events detected in Polygonoideae occurred after the Paleocene, suggesting the Beringia and North Atlantic Land Bridge may have been important routes facilitating the dispersal of Polygonoideae. Overall, we propose seven dispersal routes for Polygonoideae: Asia→North America (*Oxyria*, *Bistortia*), Asia→North America→South America (*Koenigia*), Asia→Europe (*Oxyria*, *Bistorta*, *Polygonum*, *Rumex*, *Persicaria*, *Koenigia*), Asia→Europe→North America (*Oxyria*, *Koenigia*, *Bistorta*), Asia→Africa/Asia→Europe→Africa (*Rumex*), Asia→Oceania (*Rumex*, *Persicaria*), Oceania→South America (*Muehlenbeckia*).

**Figure 6:**
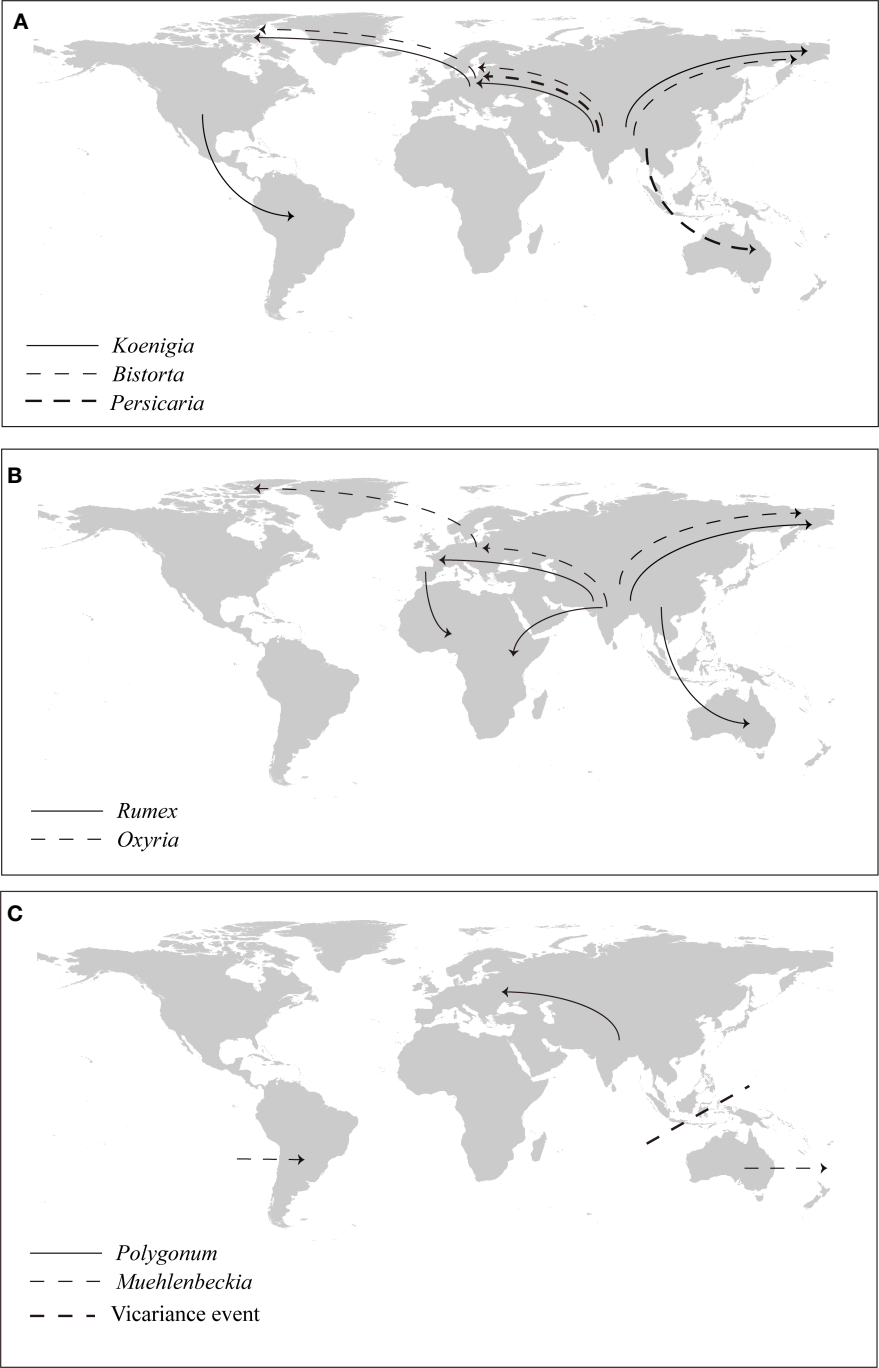
Vicariance and dispersal events in Polygonoideae. **(A)** Dispersal events in Persicarieae, **(B)** dispersal events in Rumiceae, **(C)** Vicariance and dispersal events in Polygoneae.

The Asia to North America route has been widely employed by both gymnosperm and angiosperm lineages (Wang and Ran, 2014). From the Eocene to the late Miocene, the Bering Land Bridge connected East Asia and western North America, making migration between the two continents possible (Hopkins, 1967; Tiffney and Manchester, 2001). All Asia to North America dispersal events detected in Polygonoideae happened after the Eocene (**Figures 4**, **5**), suggesting an important role of the Beringia Land Bridge as a corridor for the dispersals. The Asia to North America then to South America route could have been easily established once the spread from Asia to North America was successful, as previously reported in *Chrysosplenium* (Saxifragaceae), *Munroa* (Poaceae), *Ephedra* (Ephedraceae) and *Gunnera* (Gunneraceae) (Soltis et al., 2001; Wanntorp and Wanntorp, 2003; Ickert‐Bond et al., 2009; Amarilla et al., 2015). Similarly, long distance dispersal form Asia to Europe is achievable *via* the Himalayas to the West Pamir Mountains (Tajikistan), passing through the northern Iranian Plateau then to the Caucasus (**Figure 6**), this route was also detected in *Oryza* (Poaceae) and *Triticum* (Poaceae) (Liu et al., 2017; Spengler et al., 2021).

The Asia to Europe then to North America route was detected for species with a circumarctic distribution in Polygonoideae, after spreading from Asia to the Caucasus of Europe, species continued to spread westward to the Balkan and Carpathian Mountains, along the Alpine Mountains to the Scandinavian Mountains, finally arriving to Greenland, and then to eastern North America to attain a circumarctic distribution (**Figure 6**). A similar route to a circumarctic distribution has been detected in *Sibbaldia* (Rosaceae) (Zhang et al., 2019b). As revealed in a previous study, migration from Asia to Africa is likely to have occurred *via* the rifting of the Indian subcontinent during the Late Cretaceous/Early Paleocene (Conti et al., 2002). However, the Asia to Africa dispersals in Polygonoideae happened during or after the Miocene, ruling out ancient rifting scenarios and indicating a more recent dispersal *via* steppingstones to the Seychelles, the Comoros, and the Chagos archipelago, about halfway between Africa and Indonesia. The Miocene collision of the Afro-Arabian plate with Asia opened another channel of migration between the continents. For the Asia to Europe then to Africa route, after species spread to Europe, they migrated mainly through Mediterranean regions including the Balkans, Apennines and Iberia to Africa (**Figure 6**).

Biogeographic connections between Asia and the Southern Hemisphere are rare (Wen et al., 2014), but a series of islands including the Malay island chain and the new Guinea island chain resulting from the collision between Australia and Southeast Asia could have promoted the Asia to Oceania dispersal (2009; Hall, 2002). Generally the breakup of Gondwana can explain the disjunctive distribution pattern among New Zealand, Australia and South America (Givnish and Renner, 2004). A dispersal *via* seed dispersal related to ocean currents is more reasonable for the America dispersal in Polygonoideae.

## Conclusion

This study explored both plastome phylogenomic and biogeographic analyses of the Polygonoideae. The phylogenomic analyses revealed seven lineages corresponding to seven tribes in Polygonoideae, with Persicarieae representing the earliest-diverging lineage. The biogeographic analyses indicated Polygonoideae originated in Asia during the Paleocene, following diversification *via* long-distance dispersal and vicariance mainly after the Eocene. Generally, the results from the current study provide insights into our comprehensive understanding of the evolution, including origin, dispersal and diversification of Polygonoideae. This study also provides a good example for further study to investigate the evolution pattern of intercontinental disjunctions in a broader phylogenetic framework on a global scale.

## Perspective

Considering the extensive distribution of Polygonoideae and some still controversial relationships, e.g., the positions of Persicarieae and Oxygoneae, further sampling covering all genera and distribution ranges of Polygonoideae is necessary. Comprehensive sampling is also a basis for more accurate estimations in the timing of origin and dispersal. Additional studies focused on comparative morphology and transcriptome sequencing are needed to better understand the evolutionary relationships and history of Polygonoideae.

## Data availability statement

The datasets presented in this study can be found in online repositories. The names of the repository/repositories and accession number(s) can be found in the article/**Supplementary Material**.

## Author contributions

HZ, TD, HS, and HW designed the study. HZ, XZ, YS, and BT analyzed the data and wrote the manuscript. JL, LL, GH, JS, and TK assisted with the sampling and grammatical modifications. All authors read and approved the final manuscript.

## Funding

This study was supported by grants from the Second Tibetan Plateau Scientific Expedition and Research (STEP) program (2019QZKK0502), the Key Projects of the Joint Fund of the National Natural Science Foundation of China (U1802232), the Strategic Priority Research Program of Chinese Academy of Sciences (XDA20050203), the Youth Innovation Promotion Association of Chinese Academy of Sciences (2019382), the Ten Thousand Talents Program of Yunnan Province (202005AB160005) and Project funded by China Postdoctoral Science Foundation (2022M713333).

## Acknowledgments

We thank Peter Brownless from Royal Botanic Garden Edinburgh, Xianhui Shen from Xishuangbanna Tropical Botanical Garden and Xiaodong Li from Wuhan Botanical Garden for helping with samples collecting.

## Conflict of interest

The authors declare that the research was conducted in the absence of any commercial or financial relationships that could be construed as a potential conflict of interest.

## Publisher’s note

All claims expressed in this article are solely those of the authors and do not necessarily represent those of their affiliated organizations, or those of the publisher, the editors and the reviewers. Any product that may be evaluated in this article, or claim that may be made by its manufacturer, is not guaranteed or endorsed by the publisher.

## Supplementary material

Supplementary Figure 1
**(A)** Nucleotide diversity (pi) of 74 protein-coding genes among Polygonaceae and outgroups, genes with high nucleotide diversity (pi > 0.08) are colored in red. **(B)** Watterson’s theta (θ) of 74 protein-coding genes among Polygonaceae and outgroups.

Supplementary Figure 2 Phylogenetic tree of 54 taxa using maximum likelihood (ML) and Bayesian inference (BI) based on complete plastome sequences and remove one copy of the IR regions. Maximum likelihood bootstrap values (BS) and posterior probabilities (PP) are shown at nodes. Branches with no values listed have 100% BS and PP of 1.0.

Supplementary Figure 3 Phylogenetic tree of 60 taxa using maximum likelihood (ML) and Bayesian inference (BI) based on three chloroplast fragments (*matK*, *trnL* and *rbcL*).
