# Supplementary figures and images for: Plastome phylogenomics and biogeography of the subfam. Polygonoideae (Polygonaceae)
Source: Front Plant Sci. 2022 Oct 5;13:893201. doi: 10.3389/fpls.2022.893201 (PMC9581148; doi:10.3389/fpls.2022.893201)

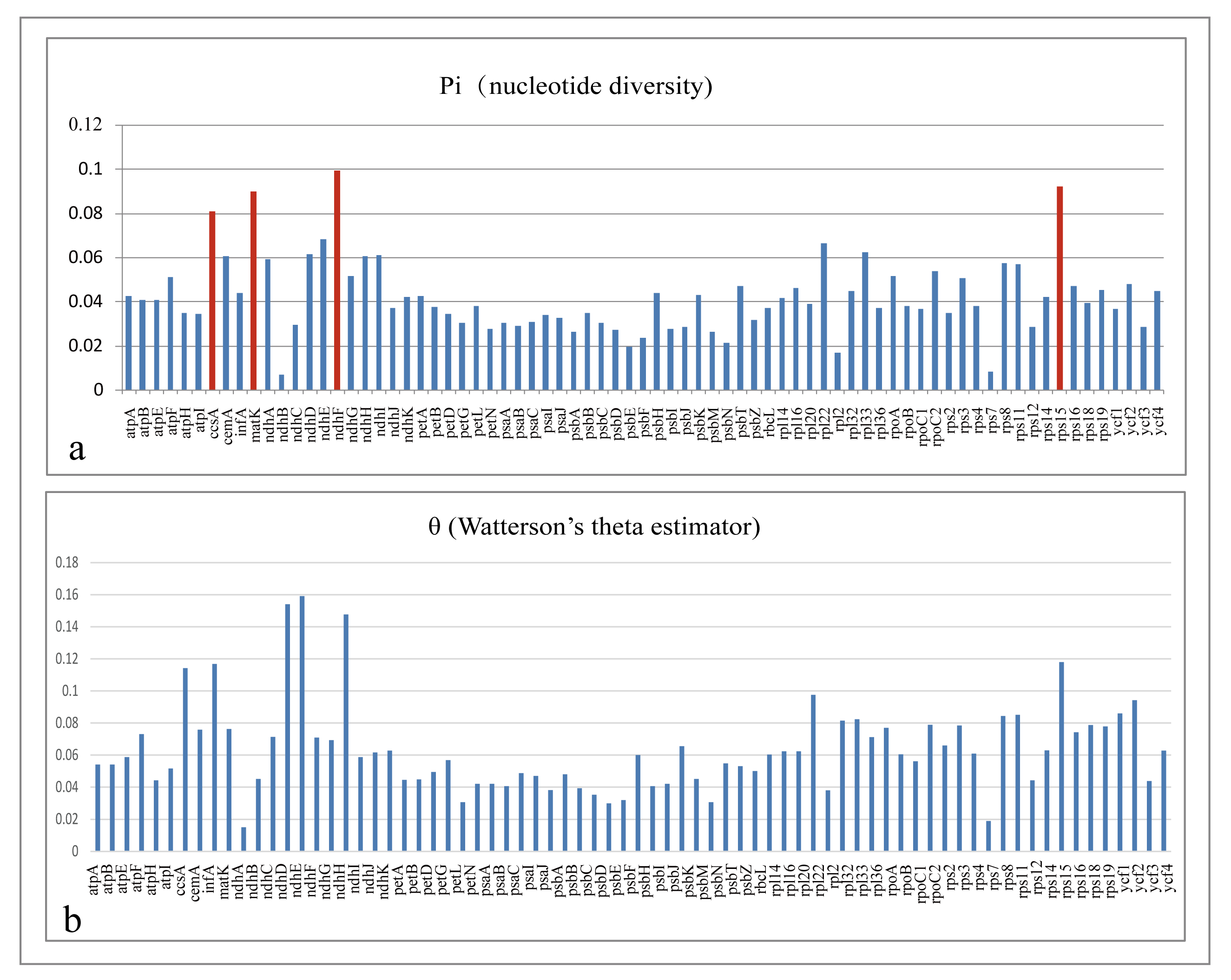

Supplement: Supplementary Figure 1 — (A) Nucleotide diversity (pi) of 74 protein-coding genes among Polygonaceae and outgroups, genes with high nucleotide diversity (pi > 0.08) are colored in red. (B) Watterson’s theta (θ) of 74 protein-coding genes among Polygonaceae and outgroups. [file Image_1.tif]

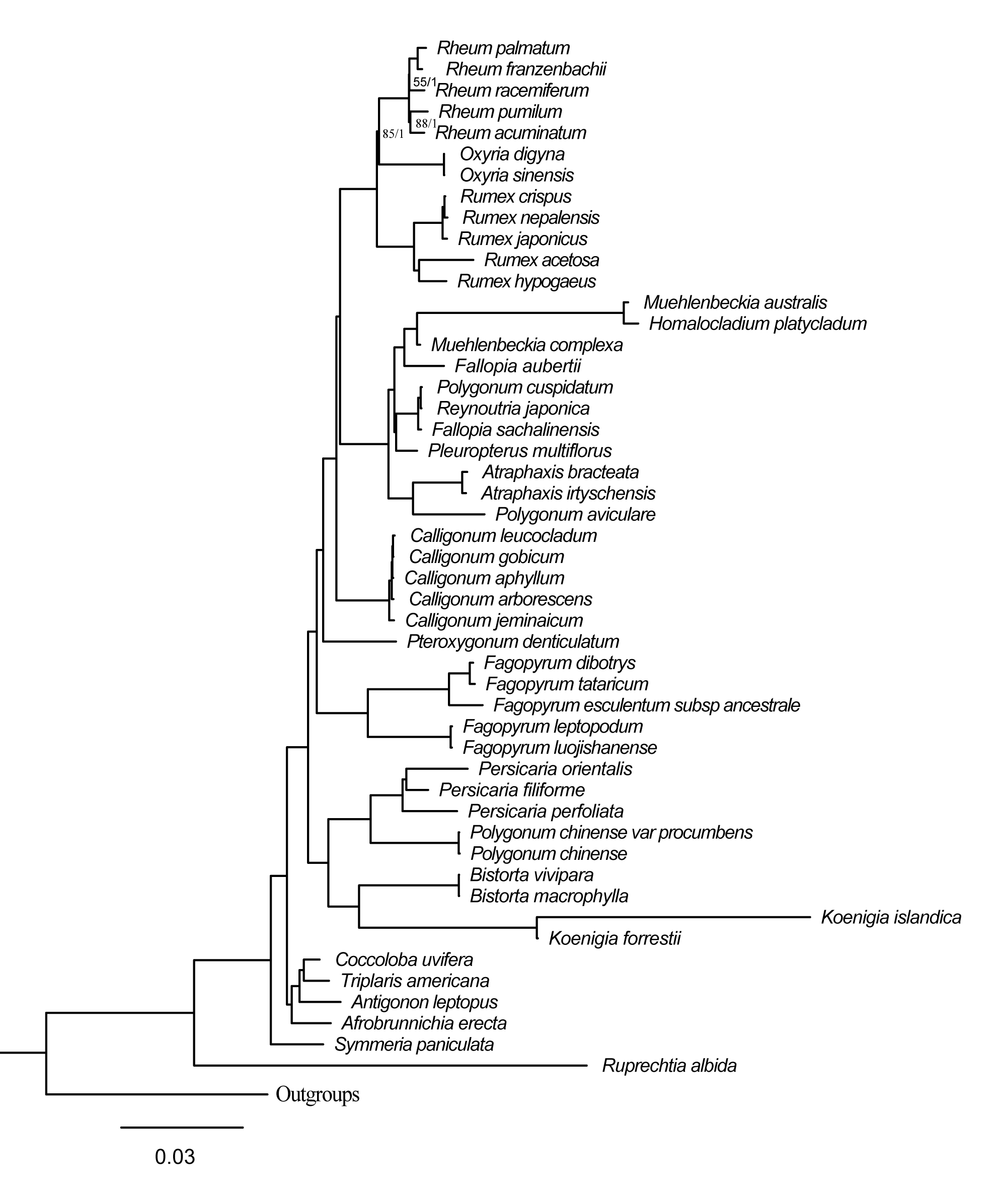

Supplement: Supplementary Figure 2 — Phylogenetic tree of 54 taxa using maximum likelihood (ML) and Bayesian inference (BI) based on complete plastome sequences and remove one copy of the IR regions. Maximum likelihood bootstrap values (BS) and posterior probabilities (PP) are shown at nodes. Branches with no values listed have 100% BS and PP of 1.0. [file Image_2.tif]

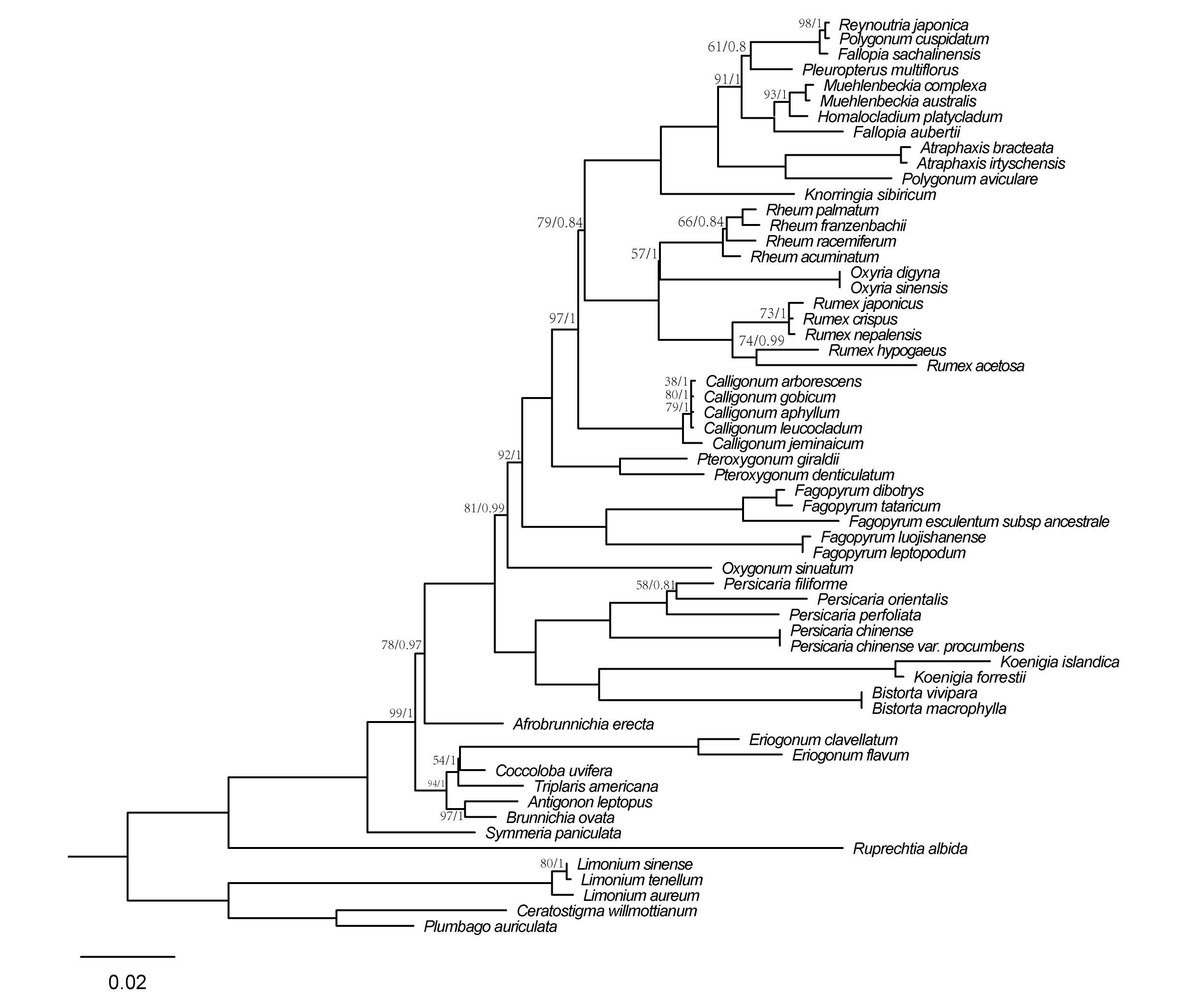

Supplement: Supplementary Figure 3 — Phylogenetic tree of 60 taxa using maximum likelihood (ML) and Bayesian inference (BI) based on three chloroplast fragments (matK, trnL and rbcL). [file Image_3.tif]
