# Supplementary material for: Plastome phylogenomics and biogeography of the subfam. Polygonoideae (Polygonaceae)
Source: Front Plant Sci. 2022 Oct 5;13:893201. doi: 10.3389/fpls.2022.893201 (PMC9581148; doi:10.3389/fpls.2022.893201)
Supplement: Supplementary file 4 [file DataSheet_1.docx]

**Supplementary Methods S1.** Discussion of fossil calibrations.

**Fossil 1**

**Name:** *Polygonocarpum johnsonii* Manchester & O’Leary

**Fossil type:** Fruit

**Source:** Manchester and O’Leary (2010)

**Locality/Stratigraphy:** Southwestern North Dakota.

**Age:** late Cretaceous (Maastrichtian), 66.0 Ma

**Assigned Node:** Crown subfam. Polygonoideae, Polygonaceae

**Discussion**: A full discussion of this fossil is presented in Magallón et al.(2015).

**Fossil 2**

**Name**: *Muehlenbeckia* sp. Pole

**Fossil type:** Partial leaf

**Source:** Pole (1992)

**Locality/Stratigraphy:** Manuherikia Group, New Zealand

**Age:** early Miocene , 66.0 Ma

**Assigned Node:** Crown genus: *Muehlenbeckia*

**Discussion:** A detailed morphological discussion of *Muehlenbeckia* fossil is presented in Pole (1992). In their study, the age of the upper boundary of Muehlenbeckia (22–19 Ma) in the early Miocene was used to calibrate the crown node of *Muehlenbeckia* as a compilation in Schuster et al. (2013).

**References**

Magallón, S., Gómez‐Acevedo, S., Sánchez‐Reyes, L.L., and Hernández‐Hernández, T. (2015). A metacalibrated time‐tree documents the early rise of flowering plant phylogenetic diversity. *New Phytol.* 207**,** 437-453. doi: [10.1111/nph.13264](https://doi.org/10.1111/nph.13264).

Manchester, S.R., and O’leary, E.L. (2010). Phylogenetic distribution and identification of fin-winged fruits. *The Botanical Review* 76**,** 1-82. doi: 10.1007/s12229-010-9041-0.

Pole, M. (1992). Early Miocene flora of the Manuherikia Group, New Zealand. 2. Conifers. *J. R. Soc. N. Z.* 22**,** 287-302. doi: 10.1080/03036758.1992.10420822.

Schuster, T.M., Setaro, S.D., and Kron, K.A. (2013). Age estimates for the buckwheat family Polygonaceae based on sequence data calibrated by fossils and with a focus on the Amphi-Pacific Muehlenbeckia. *PLoS One* 8**,** e61261. doi: 10.1371/journal.pone.0061261.
