# Supplementary material for: Plastome phylogenomics and biogeography of the subfam. Polygonoideae (Polygonaceae)
Source: Front Plant Sci. 2022 Oct 5;13:893201. doi: 10.3389/fpls.2022.893201 (PMC9581148; doi:10.3389/fpls.2022.893201)
Supplement: Supplementary file 5 [file DataSheet_2.docx]

Supplementary Table 1: Assembly information of newly sequenced plastomes of 17 Polygonaceae species

| Species | Collection number | Total reads | Assembled reads | Average coverage | Locality | Place of voucher deposition |
| --- | --- | --- | --- | --- | --- | --- |
| *Homalocladium platycladum* | zhj43 | 14369948 | 479930 | 465 | cultivated | HIB |
| *Fallopia aubertii* | zhj23 | 12997916 | 409534 | 395 | Beijing Botanical Garden | HIB |
| *Reynoutria japonica* | zhj10 | 16625400 | 656958 | 641 | Wuhan Botanical Garden | HIB |
| *Pleuropterus multiflorus* | zhj39 | 15033938 | 572820 | 554 | Wuhan Botanical Garden | HIB |
| *Polygonum aviculare* | zhj40 | 13087402 | 885010 | 846 | Henan, China | HIB |
| *Pteroxygonum denticulatum* | zhj44 | 15471838 | 1663320 | 1596 | Kumming Botanical Garden | HIB |
| *Persicaria orientalis* | zhj24 | 13247314 | 4013880 | 3956 | Beijing Botanical Garden | HIB |
| *Persicaria filiforme* | zhj03 | 15230422 | 237500 | 240 | Wuhan Botanical Garden | HIB |
| *Persicaria perfoliata* | zhj45 | 14607960 | 1106418 | 1080 | Wuhan Botanical Garden | HIB |
| *Polygonum chinense var procumbens* | zhj05 | 20520168 | 687724 | 691 | Wuhan Botanical Garden | HIB |
| *Polygonum chinense* | zhj04 | 17336940 | 208358 | 209 | Wuhan Botanical Garden | HIB |
| *Bistorta macrophylla* | zhj42 | 13974388 | 363304 | 359 | Sichuan,China | HIB |
| *Koenigia islandica* | zhj37 | 16404958 | 1493736 | 1509 | Sichuan,China | HIB |
| *Koenigia forrestii* | zhj41 | 14407576 | 720400 | 722 | Sichuan,China | HIB |
| *Coccoloba unifera* | zhj27 | 14600172 | 559712 | 518 | Xishuangbanna Tropical botanical garden | HIB |
| *Triplaris americana* | zhj29 | 13611708 | 62876 | 253 | Xishuangbanna Tropical botanical garden | HIB |
| *Ruprechtia albida* | zhj33 | 16157104 | 769102 | 770 | Royal Botanic Garden Edinburgh | HIB |

| Supplementary Table 2 Genetic characteristics of 74 protein-coding genes used in analyses, including length, nucleotide diversity (pi), Watterson’s theta estimator (θ), parsimony informative sites (PI). | | | | |
| --- | --- | --- | --- | --- |
| Gene list | Length (bp) | Nucleotie diversity (pi) | Watterson’s theta (θ) | Parsimony Informative Sites (PI) |
| *atpA* | 1524 | 0.04252 | 0.05414 | 289 |
| *atpB* | 1494 | 0.0404 | 0.05405 | 270 |
| *atpE* | 402 | 0.0406 | 0.05874 | 74 |
| *atpF* | 552 | 0.0508 | 0.07315 | 138 |
| *atpH* | 243 | 0.0349 | 0.04425 | 38 |
| *atpI* | 744 | 0.0345 | 0.05161 | 127 |
| *ccsA* | 984 | 0.0806 | 0.11426 | 334 |
| *infA* | 174 | 0.0436 | 0.07581 | 41 |
| *matK* | 1572 | 0.0898 | 0.1168 | 621 |
| *ndhA* | 1113 | 0.059 | 0.07622 | 310 |
| *ndhB* | 1530 | 0.0066 | 0.01494 | 58 |
| *ndhC* | 360 | 0.0295 | 0.04511 | 52 |
| *ndhD* | 1394 | 0.0612 | 0.07131 | 368 |
| *ndhE* | 351 | 0.0681 | 0.154 | 62 |
| *ndhF* | 2658 | 0.0991 | 0.15915 | 855 |
| *ndhG* | 531 | 0.0513 | 0.07087 | 124 |
| *ndhH* | 1185 | 0.0606 | 0.0693 | 289 |
| *ndhI* | 663 | 0.0607 | 0.14765 | 123 |
| *ndhJ* | 474 | 0.0372 | 0.0588 | 91 |
| *ndhK* | 867 | 0.0419 | 0.06164 | 186 |
| *petA* | 963 | 0.0423 | 0.0628 | 201 |
| *petB* | 645 | 0.0374 | 0.04457 | 103 |
| *petD* | 486 | 0.0343 | 0.0448 | 73 |
| *petG* | 111 | 0.0304 | 0.04942 | 19 |
| *petL* | 93 | 0.0377 | 0.05689 | 19 |
| *petN* | 87 | 0.0275 | 0.03062 | 8 |
| *psaA* | 2256 | 0.0302 | 0.04204 | 313 |
| *psaB* | 2202 | 0.029 | 0.04204 | 308 |
| *psaC* | 243 | 0.0309 | 0.04064 | 36 |
| *psaI* | 108 | 0.0337 | 0.04877 | 16 |
| *psaJ* | 134 | 0.0323 | 0.04702 | 28 |
| *psbA* | 1065 | 0.0262 | 0.03813 | 138 |
| *psbB* | 1524 | 0.0349 | 0.04809 | 249 |
| *psbC* | 1419 | 0.0304 | 0.03928 | 192 |
| *psbD* | 1059 | 0.027 | 0.03523 | 132 |
| *psbE* | 249 | 0.0192 | 0.02996 | 28 |
| *psbF* | 117 | 0.0234 | 0.03189 | 13 |
| *psbH* | 219 | 0.0436 | 0.06012 | 49 |
| *psbI* | 108 | 0.0277 | 0.04064 | 13 |
| *psbJ* | 120 | 0.0284 | 0.04206 | 18 |
| *psbK* | 183 | 0.0429 | 0.06558 | 41 |
| *psbM* | 102 | 0.0262 | 0.04518 | 11 |
| *psbN* | 129 | 0.0212 | 0.03062 | 13 |
| *psbT* | 99 | 0.0469 | 0.05486 | 21 |
| *psbZ* | 186 | 0.0316 | 0.05309 | 35 |
| *rbcL* | 1425 | 0.0368 | 0.05005 | 238 |
| *rpl14* | 372 | 0.0417 | 0.06035 | 69 |
| *rpl16* | 412 | 0.0458 | 0.06231 | 88 |
| *rpl20* | 231 | 0.0388 | 0.06231 | 57 |
| *rpl22* | 444 | 0.0662 | 0.09753 | 113 |
| *rpl2* | 829 | 0.0169 | 0.03797 | 85 |
| *rpl32* | 181 | 0.0446 | 0.08151 | 50 |
| *rpl33* | 204 | 0.0624 | 0.08229 | 50 |
| *rpl36* | 111 | 0.0369 | 0.07117 | 19 |
| *rpoA* | 759 | 0.0515 | 0.07694 | 198 |
| *rpoB* | 3219 | 0.0378 | 0.06054 | 621 |
| *rpoC1* | 2079 | 0.0364 | 0.05618 | 386 |
| *rpoC2* | 4275 | 0.0537 | 0.07888 | 1174 |
| *rps2* | 712 | 0.0349 | 0.06602 | 130 |
| *rps3* | 672 | 0.0505 | 0.07842 | 149 |
| *rps4* | 603 | 0.0377 | 0.06096 | 112 |
| *rps7* | 465 | 0.008 | 0.01888 | 26 |
| *rps8* | 402 | 0.0575 | 0.0844 | 109 |
| *rps11* | 426 | 0.057 | 0.08507 | 100 |
| *rps12* | 114 | 0.0282 | 0.04427 | 14 |
| *rps14* | 300 | 0.0419 | 0.06291 | 67 |
| *rps15* | 254 | 0.0919 | 0.11793 | 102 |
| *rps16* | 252 | 0.0468 | 0.07429 | 65 |
| *rps18* | 339 | 0.0391 | 0.07878 | 68 |
| *rps19* | 276 | 0.045 | 0.07792 | 64 |
| *ycf1* | 9925 | 0.0367 | 0.08603 | 1818 |
| *ycf2* | 9939 | 0.048 | 0.09418 | 1601 |
| *ycf3* | 507 | 0.0286 | 0.04389 | 61 |
| *ycf4* | 552 | 0.0447 | 0.06281 | 112 |

| Supplementary Table 3 List of species and sequences used in molecular phylogenetic analyses. | | | |
| --- | --- | --- | --- |
| Species | *matK* accessions | *trnL* accessions | *rbcL* accessions |
| *Knorringia sibiricum* | EU024771 | EU024789 | KT280142 |
| *Oxygonum sinuatum* | KR734898 | KR537752 | KR736970 |
| *Brunnichia ovata* | AY042561 | JQ352627 | AF297136 |
| *Eriogonum flavum* | AY042584 | AJ312350 | MG248611 |
| *Eriogonum clavellatum* | EF438000 | JQ352636 | EF437980 |
